# A Historical Essay on the Relation of Veterinary Medicine to the Medical Profession

**Published:** 1903-01

**Authors:** S. G. Burkholder

**Affiliations:** Rothsville, Pa.


### AN HISTORICAL ESSAY ON THE RELATION OF VETERINARY MEDICINE TO THE MEDICAL PROFESSION.

BY S. G. BURKHOLDER, M.D., M.D.V.,
ROTHSVILLE, PA.

WE do not possess a concise history of the remote origin of the practice of medicine or the healing art, but we have every reason to believe that Egypt is the country in which all the arts of civilized life including medicine were first cultivated with any degree of success.

Moses, in his writings, alludes to the practice of medicine among the Jews, but as far as we can learn, the privilege to practice was confined to the priests whose treatment consisted principally of promoting cleanliness and giving hygienic and spiritual advice.

The early history of medicine in Egypt is very incomplete and rather legendary. We will have to turn to Greece for the first substantial pillar upon which to base the origin and foundation of the medical and veterinary sciences of to-day.

History tells us that the Grecians were skilled in the arts and sciences eight or ten centuries before the Christian Era, and that they practiced medicine on both man and animals with marvelous results.

Chiron, of Thessaly, a descendant of the race, is recorded to have been the most skilled in the practice of the healing art, and paid equally as much attention to the equine as to the human race.

Æsculapius, another Grecian, who later, according to the legends, became the god of the healing art, and is really the founder of the modern school of medicine, was educated by Chiron and followed his footsteps. Thus we see that the patron saint of our physicians of to-day had for his preceptor a practical veterinarian as well as a practical physician. Æsculapius became a great teacher and dissected animals for the instruction of his pupils in the medical art as he practiced it.

By looking up the followers of Æsculapius we find that they all practically followed his methods for several centuries.

Among his predecessors a few gained some prominence. Among them being Erictheus, Varro, Xenophon, Calumella, Homer, Democratus, and others until we finally come down to Hippocrates who was the seventeenth or eighteenth in descent from Æsculapius. He was the most celebrated physician of antiquity, a great writer, and is to-day referred to as the father of medicine.

Thus far they all based their anatomical knowledge upon dissections of animals, and apparently gave just as much attention to the diseases of animals as to the diseases of man.

Calumella and Hippocrates wrote exhaustive treatises on the healing art as applied to animals. So you see Hippocrates might just as properly be designated father of veterinary medicine as father of medicine.

Hippocrates was so immeasurably superior to his contemporaries that it seemed to have acted as a check to further attempts at improvement for several centuries.

No real progress was made, especially in anatomy, owing to the researches being confined to animals, until the time of Erasistratus, about 250 B.C., who was the first to dissect human bodies.

Henceforth up to the beginning of the Christian Era no perceptible advancement is evident.

During the first part of the Christian Era the dissection of human subjects was forbidden under heavy penalties. The medical profession was divided into four or five differents sects who were constantly disputing with one another and no material progress was made.

During the latter half of the second century of the Christian Era, Galen, a very celebrated physician and great writer, loomed into prominence. He was a great anatomist and in his studies he dissected apes as being the most like human subjects.

For the next thousand years the advancement in the art of medicine was very slow. In fact, Galen reigned supreme throughout the civilized world until within the last three hundred years.

No human subjects were openly used for dissection until the time of Frederick II., King of Sicily, about 1200 A.D. He passed a law prohibiting anyone from practicing surgery without having first acquired some knowledge of anatomy by dissecting human bodies. His example was followed by others throughout the civilized nations. Thus it appears that up to the time of the Christian Era the art of healing was studied and practiced for the relief of the brute creation as well as of man, all being treated on the same principle by the same physicians.

About 300 A.D. Vegetius, also a Greek disciple of Chiron, collected and revised all the works on the art of healing animals that had been published up to that time.

We find no evidence that the practice of veterinary medicine existed as a distinct science previous to this time, but we do find that those versed in the art of healing who principally confined their efforts to the care and treatment of the horse held honored positions and recognized ranks in the Roman army several centuries before the beginning of the Christian Era. The horse was an indispensable factor in the art of warfare and those who looked after the medical needs of this noble animal had conferred upon them the foremost titles and honors of the land. This appreciation of the services of the veterinarian was not confined to the Roman Empire, but later the French, the Normans, and the English held them in high esteem and conferred upon them similar titles of honor.

The early history of medicine seems to prove that the original pioneers of the healing art and their followers treated all ailments of both man and beast with equal consideration and skill. The two distinct professions as they appear to-day, originated together, grew up together, were advanced and amplified by the same men, and were one and inseparable for a period of at least 1000 years. After the dissection of human bodies by the physicians and their students received legal sanction, and various medical schools were established, the followers of the medical practice confined themselves to the human family more and more completely until finally the poor beast was apparently dropped from their consideration altogether, so far as investigating and treating their diseases was concerned.

The practice of the healing art as applied to humanity became a distinct branch of science and grew gradually but slowly until the latter part of the eighteenth and beginning of the nineteenth century. The nineteenth century may be considered as the epoch of physiological research and clinical observation.

We are getting ahead of our story. We left the animal creation uncared for, from a medical point of view, in the early part of the Christian Era. They were neglected by those best skilled in the art of ministering to their ailments and fell into the hands of an army of ignorant and superstitious rubbish.

Henceforth for several hundred years might be termed the dark age of the veterinary science. But thanks to an omnipotent Providence, during the latter half of the eighteenth century, out of the oblivious sea of illiteracy and superstition the veterinary profession has once more sprung and with advancing steps has displayed energy, perseverance, and skill, until to-day it is again working on a common plane with the medical profession.

The position held by the veterinarian and the duty he should strive to perform should not redound simply to the economic advantages of the stock owner; but his aim should be to annihilate diseases from the lower animals many of which are transmissible directly to man, thus preventing transmission of contagious and parasitic diseases and protecting human life. This is far more important than the treatment of disease.

While the physician is the alleviator of disease, the veterinarian is the preventer of its occurrence. He is the safeguard to public health. The responsible position held by the veterinarian is not appreciated by the laity, nor even by many physicians, but the time is not far distant when his real value will be recognized and he will be an indispensable factor to every community and work in harmony with the physician in his efforts to relieve suffering and save human lives. The veterinary profession should be and will be represented in every town, city, county, State, and national board of health. The amount of physical suffering and death in man due to direct transmission from corresponding diseases among our domestic animals is not yet universally realized. Every medical school worthy of the name will in the near future include in its college curriculum a chair of comparative pathology and comparative medicine. Then, and not until then, will the exalted position of the veterinarian be generally recognized.

Let us enumerate a few of the more important diseases common to both animals and man and thus bring out more strongly the relation the veterinarian bears to the medical profession.

1. Anthrax, though not so common now as formerly, may be transmitted to man through an abraded surface of the skin or mucous membrane. It gives rise to a local lesion at the seat of inoculation. It may form a papule, rapidly becoming a vesicle, form a scab and dry off in a few days. It may become pustular, surrounded by an inflammatory and indurated area giving rise to very distressing symptoms, both local and constitutional, and lead to a large slough, and the patient recover. Or the infection may become general and the patient succumb. Thanks to the advancement of the veterinary science the disease is kept under control and will finally be annihilated by means of successive animal inoculations of an attenuated virus.

2. Actinomycosis is another disease sometimes found in man. This is usually transmitted directly or indirectly from diseased cattle. The discharge from the local abscess on the animal's jaws may come in contact with an abraded surface or mucous membrane of man and give rise to the characteristic local lesion. Stablemen are often in the habit of chewing the end of a straw while in the pursuit of their duties, which, should it happen to be contaminated with the virus, may be the means of inoculating the man. It is an undecided question whether or not the consumption of meat from animals afflicted with this disease has ever been the source of the transmission.

3. Glanders, a disease equally fatal to both the human and equine race, is at present rather rare in man. This is due to the fact that the veterinarian recognizes it in the horse in the early stages and the animal is properly isolated and destroyed, which prevents further infection.

4. Tuberculosis, the destroyer of thousands of human lives every year, is too well known to need any discussion in this article. Suffice it to say the only way to lessen its career of destruction is by a concerted and harmonious effort of both the veterinary and medical professions. The disease is undoubtedly transmissible from animals to man (Professor Koch's theory notwithstanding) and the only way to get it under control is to prevent as far as possible every source of infection. This is not an easy matter and will take many years to accomplish. Both professions will have to be thoroughly organized, for our enemy the tubercle bacilli are a stealthy and treacherous foe. They do not attack us with the sound of the trumpet and roll of the drum, but swoop down upon us in darkness and in silence, and suddenly appear in our midst when least expected. Owing to the latency of the disease they provoke and the absence of outward manifestations, they are a potent factor in the propagation of the infection and make a most formidable antagonist.

The only way to subdue the enemy is to organize a powerful garrison of physicians thoroughly disciplined and strongly fortified to vigorously fight the enemy already in our midst, and prevent reinforcements from the bovine and other nations of the animal tribe by a dense line of vigilant veterinary pickets.

Some of the parasites causing disease in animals require man as a host before they can complete their cycle of existence, and very unwelcome guests they prove to be. Among the parasitic enemies may be mentioned the cysticercus bovis and the cysticercus cellulosa, who, if they gain access to the alimentary canal of man unharmed will develop into the tænia saginatta and tænia solium, respectively. These, while they do not cause the death of their hosts directly, give rise to a great deal of discomfort and reduce his general strength and resisting powers, so that he yields more readily to unfavorable influences which, if not relieved, will shorten his life indirectly.

Another parasite more destructive than either of the above two occasionally finds man a very unwilling but submissive entertainer. This is the trichina spiralis. We usually find them in the cystic form in the nucleus of the pig where they remain, and if their host is allowed to live long enough they finally die and undergo calcareous degeneration. If, on the other hand, the host is killed while the parasite is still alive and his carcass is consumed by man, they either succumb to the excessive heat the pork is subjected to before it is eaten, or if they escape that ordeal, they gain their liberty in the alimentary tract of their second host or man. Here they develop, cohabit, propagate, and die, leaving a small army of young parasites, who at once begin to migrate to the muscular portion of the human body. This process of migrating causes the host an unendurable amount of agony, to which he often succumbs.

Here, again, we find our benefactor the veterinarian on picket duty, carefully guarding the approach of the parasitic enemy with fixed bayonets ready to slay the death-dealing foe thus preserving the comfort, if not saving the life, of his fellow man.

The inspection of animals in the public stockyards and abattoirs by the Bureau of Animal Industry is instrumental, no doubt, in saving the lives of a number of people annually by preventing diseased meats from being consumed by the general public and prohibiting diseased animals from being allowed to mingle with healthy animals and man, thus spreading the disease.

The system is defective only in that it is not extensive enough, which is due principally to the lack of legislative support.

All animals whose carcasses are used for human food should pass a rigid ante-mortem and post-mortem inspection, and those which are kept for their milk, breeding, and other purposes should be thoroughly inspected by skilled veterinarians at stated periods, say every six months, and certificates of soundness should be issued for each animal thus inspected and passed as sound. In this way tuberculosis and kindred diseases could be finally blotted out among animals, and by strictly observing the laws of hygiene it would be a comparatively easy matter for the medical profession to exterminate some of these diseases among the human family. Without the assistance and co-operation of the skilled veterinarian backed by legislative support, these transmissible diseases can never be eradicated. It is only by the united efforts of the veterinarian and medical professions that these results may be accomplished.

The significance of the veterinarian's position as a preventer of disease among man and animals, and his intimate relation to the medical profession will become more evident year by year until finally history will repeat itself and the two medical sciences so closely related will be merged into one, when all prospective practitioners will be educated in the same school of medicine, the veterinary science simply becoming a branch of general medicine.

